# Multi-Modal Investigation of Metabolism in Murine Breast Cancer Cell Lines Using Fluorescence Lifetime Microscopy and Hyperpolarized 13C-Pyruvate Magnetic Resonance Spectroscopy

**DOI:** 10.3390/metabo14100550

**Published:** 2024-10-15

**Authors:** Sarah Erickson-Bhatt, Benjamin L. Cox, Erin Macdonald, Jenu V. Chacko, Paul Begovatz, Patricia J. Keely, Suzanne M. Ponik, Kevin W. Eliceiri, Sean B. Fain

**Affiliations:** 1 Morgridge Institute for Research, 330 N. Orchard St., Madison, WI 53715, USA; sarah.erickson-bhatt@marquette.edu (S.E.-B.); blcox@uchicago.edu (B.L.C.); eliceiri@wisc.edu (K.W.E.); 2 Laboratory for Optical and Computational Instrumentation, University of Wisconsin at Madison, 1675 Observatory Dr., Madison, WI 53706, USA; 3 Department of Cell and Regenerative Biology, University of Wisconsin at Madison, 1111 Highland Ave., Madison, WI 53705, USA; ponik@wisc.edu (S.M.P.); 4 Department of Medical Physics, School of Medicine and Public Health, University of Wisconsin at Madison, 1111 Highland Ave., Madison, WI 53705, USApaul.begovatz@outlook.com (P.B.); 5 Department of Radiology, Duke University Medical Center, 2424 Erwin Road, Suite 302, Durham, NC 27705, USA; 6 Department of Biomedical Engineering, University of Wisconsin at Madison, 1550 Engineering Dr., Madison, WI 53706, USA; 7 Department of Radiology, Carver College of Medicine, University of Iowa, 200 Hawkins Drive, Iowa City, IA 52242, USA

**Keywords:** breast cancer, metastatic potential, metabolism, FLIM, MRS, hyperpolarized, carbon-13

## Abstract

**Background/Objectives**: Despite the role of metabolism in breast cancer metastasis, we still cannot predict which breast tumors will progress to distal metastatic lesions or remain dormant. This work uses metabolic imaging to study breast cancer cell lines (4T1, 4T07, and 67NR) with differing metastatic potential in a 3D collagen gel bioreactor system. **Methods**: Within the bioreactor, hyperpolarized magnetic resonance spectroscopy (HP-MRS) is used to image lactate/pyruvate ratios, while fluorescence lifetime imaging microscopy (FLIM) of endogenous metabolites measures metabolism at the cellular scale. **Results**: HP-MRS results showed no lactate peak for 67NR and a comparatively large lactate/pyruvate ratio for both 4T1 and 4T07 cell lines, suggestive of greater pyruvate utilization with greater metastatic potential. Similar patterns were observed using FLIM with significant increases in FAD intensity, redox ratio, and NAD(P)H lifetime. The lactate/pyruvate ratio was strongly correlated to NAD(P)H lifetime, consistent with the role of NADH as an electron donor for the glycolytic pathway, suggestive of an overall upregulation of metabolism (both glycolytic and oxidative), for the 4T07 and 4T1 cell lines compared to the non-metastatic 67NR cell line. **Conclusions**: These findings support a complementary role for HP-MRS and FLIM enabled by a novel collagen gel bioreactor system to investigate metastatic potential and cancer metabolism.

## 1. Introduction

Breast cancer is the second leading cause of cancer death in women in the U.S. [1]. Primary breast cancer itself is not deadly; it is only when the cancer spreads to other vital parts of the body (i.e., it metastasizes) that it becomes lethal. Metastatic breast cancer tends to travel to the lungs, bones, and brain, among other areas. However, not all breast tumors are metastatic. Additionally, some tumors have a tendency to spread cells to other areas of the body, and then those cells remain dormant for a period of time (a year to several decades) before they escape from dormancy and form secondary tumors [2,3]. These are referred to here as metastatically dormant. During primary breast surgery (typically a lumpectomy or mastectomy), the sentinel axillary lymph nodes are checked and if they are found positive for cancer, then metastasis has likely occurred and additional treatment such as radiation or chemotherapy is recommended. However, many patients who are diagnosed at an early stage, with small tumors and no indication of regional lymph node metastases, can still have a high level of recurrence (>25% to 30%) after more than 10 to 15 years [4]. 

Current clinical imaging methods cannot determine which breast tumors will become metastatic or metastatically dormant and which are not metastatic at all. This study utilizes a unique previously reported in vitro platform [5] for investigating image markers of metabolism at multiple spatial scales in murine breast cancer cell lines from metastatic and metastatically dormant to non-metastatic breast tumors cultured in 3D collagen hydrogels mimicking the tumor extra-cellular matrix (ECM). Metabolic signatures are measured using two different methods, at the cellular scale using fluorescence lifetime imaging microscopy (FLIM) and at the whole-tumor scale using hyperpolarized (HP) [1-13C] pyruvate MR spectroscopy (HP-MRS). By imaging metabolism at multiple scales and within an ECM that mimics the tumor microenvironment, we are able to compare signatures at both the cellular level (microscopy) and the clinical level (noninvasive whole-sample imaging).

Normal tissues in the presence of oxygen prefer oxidative phosphorylation to metabolize glucose to pyruvate by glycolysis, and most of this pyruvate is then oxidized to CO_2_ in the mitochondria via the tricarboxylic acid cycle. However, in the absence of oxygen, cells normally undergo anaerobic glycolysis where most of the pyruvate generated by glycolysis is converted to lactate. However, cancer cells are known to undergo aerobic glycolysis and convert most glucose to lactate whether or not oxygen is present, a phenomenon first discovered by Otto Warburg and termed the Warburg Effect [6,7]. HP [1-13C] pyruvate has been used in several tumor models including sarcoma, glioblastoma, lymphoma, and prostate, demonstrating elevated levels of lactate in the tumor regions compared to normal tissue [8,9]. Conversion between pyruvate and lactate occurs by the reaction:

pyruvate + NADH + H^+^ ⇔ lactate + NAD^+^

facilitated by lactate dehydrogenase (LDH). The ratio of lactate-to-pyruvate in vivo has been used as a surrogate measure of metabolism along the glycolysis pathway and to calculate the cytosolic ratio of the oxidized to reduced form ([NAD+]/[NADH]) of nicotinamide adenine dinucleotide (NADH) [10]. 

The rate of 13C-label exchange between the injected pyruvate and the endogenous lactate pool will be influenced by the delivery of pyruvate to the tissue via perfusion, the expression of LDH and the monocarboxylate transporters (MCTs) that move pyruvate into the cytosol, and the intracellular concentrations of pyruvate and lactate and the coenzymes NADH and NAD+, all of which will determine the activity of LDH in the cell [11]. Specific to breast cancer, the factors influencing the lactate/pyruvate ratio appear to vary by tumor type. For example, hyperpolarized 13C-labeled pyruvate MRS studies of T47D (Luminal A) human breast cancer cells showed that MCT1 was rate-limiting [12], whereas in two murine breast cancer models, lactate labeling was correlated with total LDH activity in the tumor but not MCT1 expression [13]. In experiments on cells from tumors with differing malignancies, triple-negative breast cancer cells (MDA-MB-231) showed lower lactate labeling than hormone receptor-positive cells (MCF-7 cells) [14]. Lactate labeling in these cells was shown not to depend on MCT or LDH expression but on the glucose and glutamine concentrations in the cell media. In a similar comparison of highly metastatic (4T1) and metastatically dormant murine breast cancer models (4T07), lactate labeling was slightly (but not significantly) higher in the highly metastatic model [13]. The complexity of these findings speaks to the need for an improved understanding of breast cancer cell metabolism and the impact of the multiple influences on measured hyperpolarized 13C-labeled pyruvate MRS in the translational setting. 

Advances in multiphoton microscopy (MPM) also enable interrogation of the breast tumor microenvironment and metabolism at the cellular scale [15]. Both the fluorescence intensity and the temporal decay of fluorescence are used in FLIM. MPM has been used to image endogenous fluorophores in breast tissue including tryptophan, flavin adenine dinucleotide (FAD), NADH and nicotinamide adenine dinucleotide phosphate (NADPH)—hereafter NAD(P)H since the two forms cannot be distinguished with endogenous fluorescence—and endogenous second-harmonic-generation (SHG) signals from fibrillar collagen [16]. Longer FAD and NAD(P)H lifetimes, i.e., the time for the fluorophore to return to its ground state after excitation, indicated the presence of ductal carcinoma in situ in fixed histopathological sections of human breast tissue [17]. The metabolic basis for these findings is that FAD acts as an electron carrier in the production of ATP through oxidative phosphorylation, while NADH carries electrons in the production of ATP during glycolysis. The intensity and time decay of both molecules are sensitive to the immediate chemical environment due to their concentration and fluorophore enzymatic states with longer decay attributed to the enzymatically bound fractions. Since NAD(P)H and FAD each represent a different redox state, quantification of their intensity ratio is a surrogate of cell and tissue chemical redox conditions. Concentration-based redox ratios have been used to differentiate normal from cancerous tissue as well as levels of cancer progression and response to treatment [18,19,20,21,22,23,24,25,26,27].

In this study, we combine the HP-MRS and FLIM methods using a novel bioreactor design that is compatible with optical microscopy and MRS to analyze the metabolism of murine breast cancer cell lines in 3D collagen cell cultures [5]. The main hypothesis of this work is that controlled studies with HP-MRS will show differences in the lactate/pyruvate ratio with the breast cancer metastatic potential of the cell lines. A secondary hypothesis is that independent measures of cellular-scale FAD and NAD(P)H intensities and lifetimes using FLIM will correlate with metastatic potential and the lactate/pyruvate ratio measured with HP pyruvate MRS, thus providing a complementary assay that can facilitate the interpretation and translation of these methods toward the understanding of metabolism in clinical breast cancer studies.

## 2. Methods

### 2.1. Cell Culture in the Bioreactor

**Figure 1:**
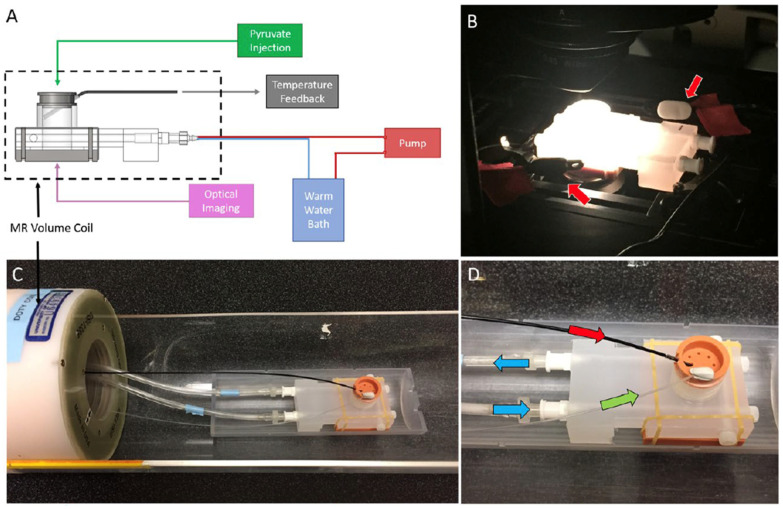
(**A**) The multi-modal bioreactor design uses a 3D collagen gel cell culture in the MRI-compatible bioreactor chamber with (**B**) a transparent portal in the base that can be placed on a fluorescence microscope stage for fluorescence lifetime imaging microscopy (FLIM setup). (**C**) The bioreactor system in the MRI setup adjacent to the volume coil was used for signal excitation and detection. (**D**) Culture media flow was constantly maintained (green arrow) with temperature control maintained by water bath flow around the culture volume (blue arrow). Hyperpolarized metabolic substrates (13C1-pyruvate in this case) were injected via bolus infusion into base of the culture chamber (red arrow).

The materials consisted of highly metastatic (4T1, CRL 2539), metastatic-dormant (4T07, CVCL B383) and non-metastatic (67NR, CVCL 9723) murine breast tumor cell lines, previously characterized by Aslakson et al. [28], which were purchased from ATCC. The cells were cultured in RPMI 1640 media with 10% fetal bovine serum at 37 °C with 5% CO_2_ in a 3D collagen gel bioreactor system. The 3D bioreactor system was designed for both FLIM and HP-MRS, as shown in Figure 1 [5]. A detailed description of the bioreactor design is provided in [5]. Briefly, the MR-compatible device contained an optical window to enable sequential imaging of the same collagen gel using multiphoton FLIM and HP-MRS. The bioreactor contains ports for temperature monitoring, injection of contrast agents, and heated water flow for temperature control.

For all experiments, cells were cultured in 3D collagen gels (1.5 mL) prepared on the day of imaging at a concentration of 8 million cells/gel with a final gel concentration of 2 mg/mL [29]. FLIM and HP-MRS were performed in the same gels, on the same imaging day. The collagen gels were prepared and polymerized directly in the bioreactor chamber, and 9 gels (3 of each of the 4T1, 4T07, and 67NR cell lines) were imaged sequentially via FLIM and HP-MRS on the same day.

### 2.2. Fluorescence Lifetime Imaging Microscopy

Was performed the experiment at the microscale using a custom-built MPM. The laser illumination was provided by a wavelength-tunable Mai-Tai Deep See Ti:Sapphire laser (Spectra Physics, Palo Alto, CA, USA) tuned to 740 nm (with a 450/70 nm filter) to image NAD(P)H and 890 nm (with a 562/40 nm filter) to image FAD. Photons were detected by a Gallium Arsenide Phosphide photomultiplier tube, H7422P-40 (GaAsP-PMT; Hamamatsu Photonics, Hamamatsu, Japan). WiscScan software (LOCI, UW-Madison, v.7.3) was used to drive the scanning and data acquisition. Time-correlated single-photon counting (TCSPC) was performed using an SPC-830 photon counting board with DC-100 control electronics (Becker and Hickl, Berlin, Germany). Each fluorescence lifetime image was collected for 150 s using a 20× VC air objective (Nikon USA, Melville, NY, USA).

**Figure 2:**
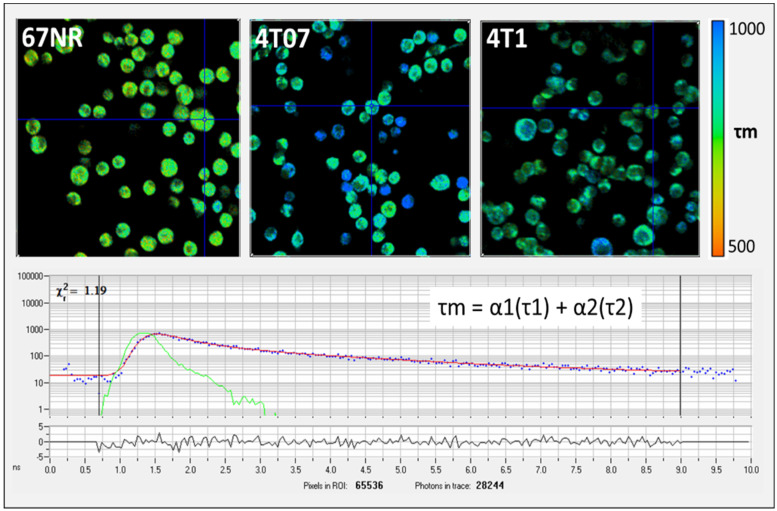
(**Top**) Example fluorescence lifetime imaging microscopy (FLIM) images showing mean lifetime (τ_m_) for NAD(P)H from non-metastatic (67NR), metastatically dormant (4T07), and metastatic (4T1) murine breast cancer cells (**upper images**). Cells were imaged at a 740 nm wavelength with a 450/70 nm filter. The color-coded images in the first row (**upper images**) show that the 67NR cells have a shorter τ_m_ (more yellow in color) compared to the longer τ_m_ in 4T07 and 4T1 cells (more blue in color). The cross hairs centered on specific cells in each panel indicate where fluorescence life time measurement is localized. (**Bottom**) Example photon lifetime distribution from a single pixel location as displayed by the SPCImage software (v8.0). Units are in nanoseconds (ns).

Free and bound NAD(P)H can be separately imaged by FLIM [30]. For each FLIM imaging session, three measurements were collected at different locations within the gel and averaged, and one HP-MRS measurement was collected for the entire gel. The FLIM data were fitted and analyzed using SPC Image (Becker and Hickl). The lifetime data were analyzed using SPCImage software (v8.0) to generate color maps of the weighted average of the parameter of interest (τm, τ1, τ2, α1, α2) fit for a two-term exponential model [30]. The average lifetime component (τ mean, or τm) is calculated by τm = α1 × τ1 + α2 × τ2, where τ1 and τ2 are the short- and long-lifetime components, respectively, and α1 and α2 are the fractional contribution of each lifetime component (Figure 2). In this work, the redox ratio is calculated from the intensity data as FAD/(FAD + NAD(P)H).

### 2.3. Hyperpolarized Pyruvate MRS

Dynamic nuclear polarization (DNP) was used to transfer polarization from electron spins of paramagnetic centers in a glassy frozen solution to neighboring nuclear spins [31]. The 13C-labeled compound (>99% neat [1-13C]-Pyruvic Acid (PA)) was mixed with a free radical in a glassing solvent to produce an amorphous solid when cooled to ~1K inside a 3.35T magnet. PA is commonly used for HP MR by dissolution DNP because it is a highly concentrated, self-glassy compound, and has a long T1 relaxation time [32]. Specifically, 30 µL of [1-13C] PA (Cambridge Isotope Laboratories Inc., Tewksbury, MA, USA) and 15 mM trityl radical (Ox063, GE Healthcare, Little Chalfont, UK) was polarized at 1.4K in a Hypersense polarizer (Oxford Instruments, Abingdon, UK) for ~1 h. A total of 4 mL of solvent containing 1.2 mL 426 mM NaOH, 1.4 mL 400 mM Tris buffer and 1.4 mL 250 mg/L EDTA was used to dissolute the sample. The [1-13C] PA was drawn off and rapidly injected into the sample volume of the bioreactor. A dual-tuned 1H/13C volume coil (Doty Scientific, Columbia, SC, USA) was used to acquire dynamic global spectra (flip angle = 10°, repetition time [TR] = 3000 ms) with transmit and receive frequencies centered on the expected pyruvate resonance. Ratios of lactate to pyruvate (Lac/Pyr) were calculated from the Gaussian fit of the respective peak areas using prior knowledge and the AMARES algorithm within the jMRUI v5.2 software package [33], after zero-filling (4096) and the summation of the entire time course of the HP-MRS spectra.

### 2.4. Data and Statistical Analysis

The study was powered based on pilot studies with FLIM; a statistician was consulted to determine the sample size (N = 9; 3 cultures per cell line) required to achieve the sufficient power for significance, assuming alpha = 0.05 and a power of 0.8. The HP-MRS results were intended to inform us on which FLIM measures are of greatest relevance to glycolytic vs. oxidative phosphorylation. Results were represented as mean ± SD over the 3 repeated cell cultures, and differences between groups were assessed using ANOVA and Tukey’s honestly significant test, with *p* ≤ 0.05 considered to be the threshold for statistical significance. Spearman correlations were used for comparing associations between measures and across methods.

## 3. Results

**Figure 3:**
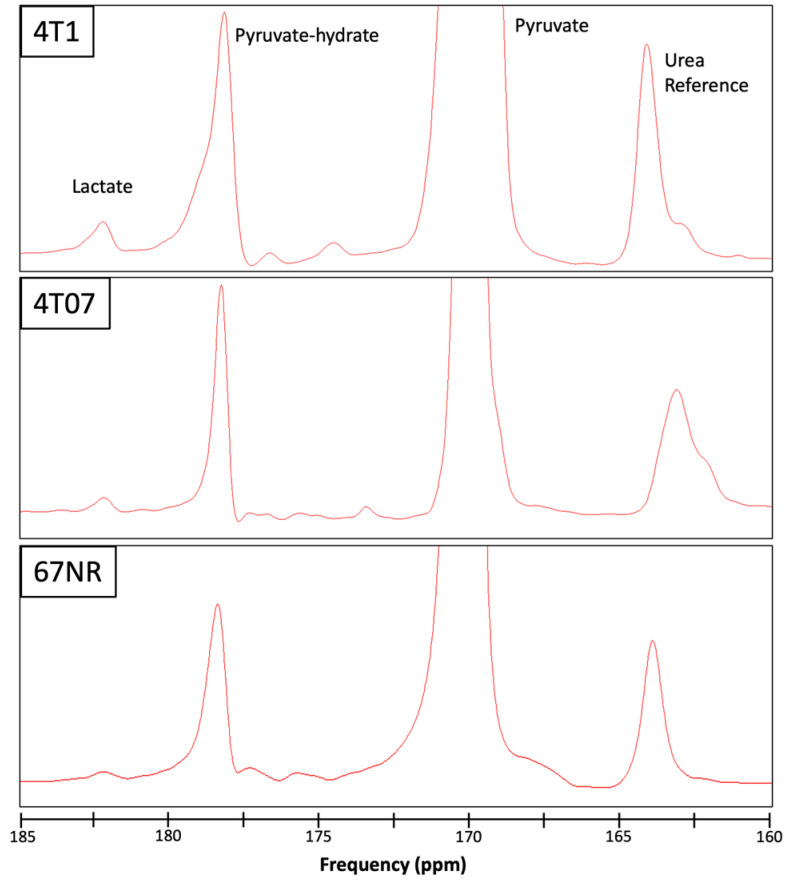
Representative spectra from the 3 cell lines studied, 4T1 (**top**), 4T07 (**middle**), and 67NR (**bottom**), showing the pyruvate substrate (170 ppm, truncated), pyruvate hydrate (178 ppm) and lactate (182 ppm) components of hyperpolarized [1-13C] pyruvate metabolism. A vial of urea was included adjacent to the bioreactor chamber as a reference (163 ppm) for calibration. Elevated lactate is apparent for the 4T1 (highly metastatic) in contrast to the 4T07 and 67NR (non-metastatic) cell lines. Note that the small peak near the lactate frequency for the 67NR spectra was measured to be below the noise threshold by the jMRUI analysis software (v5.2).

**Figure 4:**
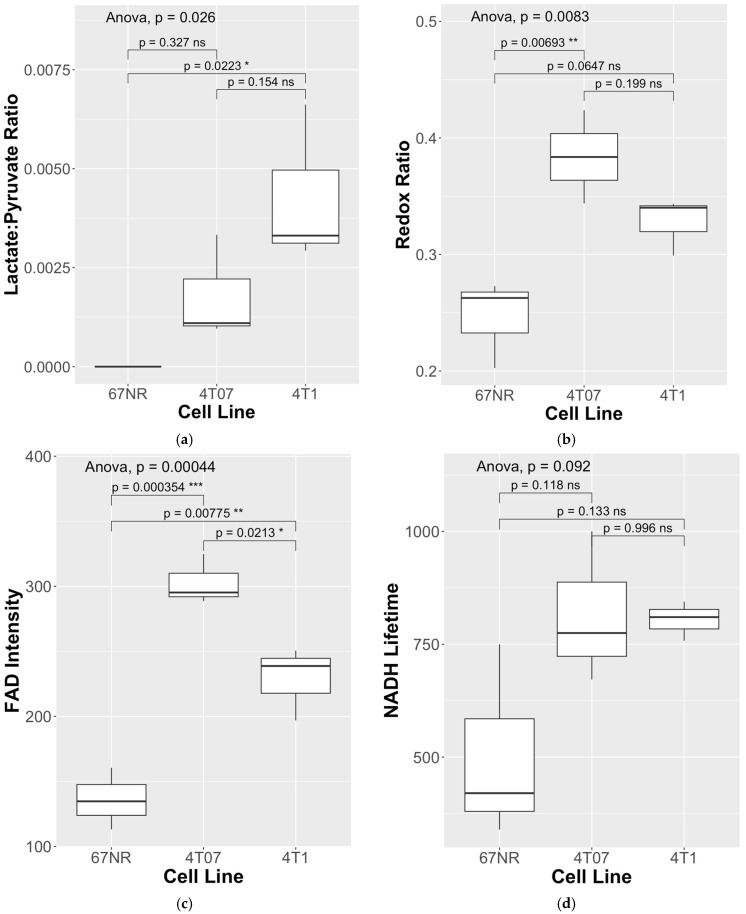
(**a**) An elevated lactate/pyruvate (Lac/Pyr) ratio was found for the 4T1 (highly metastatic) vs. 67NR (non-metastatic) cell lines, in contrast to (**b**) where the elevated redox ratio and (**c**) FAD intensity was found for 4T07 (metastatic-dormant) vs. both the 4T1 (highly metastatic) and 67NR (non-metastatic) cell lines. (**d**) NADH lifetimes trended higher for both 4T07 (metastatic-dormant) and 4T1 (highly metastatic) cell lines compared to the 67NR (non-metastatic) cell line. Three replicates were performed in parallel for each cell line, for a total of N = 9 data points per cell-line. * indicates statistical significance at *p* < 0.05, ** *p* < 0.01, *** *p* < 0.001, and ns stands for “not significant”.

To assess differences in metabolism across three triple-negative breast cancer (TNBC) cell lines (4T1, 4T07, and 67NR) with different metastatic potential, cells were cultured within 3D collagen gels in the bioreactor system (Figure 1). Endpoints for both HP-MRS and FLIM are summarized in Table 1 for each of the 3 replicates per cell line. Representative images for the mean lifetime (Tau Mean) for NAD(P)H are shown for 67NR, 4T07, and 4T1 cell lines in Figure 2. Lactate production was below the noise threshold for the 67NR (non-metastatic) cell line but was detected for the 4T07 (metastatic dormant) and 4T1 (metastatic) cell lines (Figure 3) with the Lac/Pyr ratio increasing with increasing murine breast cancer cell metastatic potential (Figure 4a). Lac/Pyr for the highly metastatic 4T1 cell line was significantly greater than that for the non-metastatic 67NR cell line (*p* = 0.02) but not for 4T1 vs. 4T07 (4.3 × 10^−3^ vs. 1.8 × 10^−3^, *p* = 0.15).

The redox ratio (Figure 4b) was significantly elevated in the metastatic-dormant 4T07 cell line vs. the non-metastatic 67NR cell line (0.384 vs. 0.246, *p* < 0.01). The redox ratio for the 4T1 (highly metastatic) cell line was qualitatively higher than that for the 67NR cell line (0.327 vs. 0.246) but not significantly different from either that of the 67NR (*p* = 0.07) or the 4T07 (*p* = 0.2) cell line. The FAD intensity was significantly different between all three cell lines. FAD intensity was elevated in the 4T07 vs. the 67NR cell line (302.9 vs. 136.1, *p* < 0.01) and in the 4T07 (metastatic-dormant) vs. the highly metastatic 4T1 cell line (302.9 vs. 228.7, *p* = 0.021). FAD lifetime (τm) was significantly longer in the 4T07 cell line compared to that in 67NR (948.9 vs. 715.1, *p* < 0.001) and 4T1 (948.9 vs. 854.3, *p* = 0.021) (Figure 4c). The FAD intensity seems to have driven the pattern observed for the redox ratio (Figure 4b, Table 1) since the NAD(P)H intensity did not differ between any cell line (ANOVA *p*-value = 0.95). There was a trend toward greater NAD(P)H lifetime for both metastatic cell lines compared to the non-metastatic 67NR cell line (Figure 4d), though neither was significantly different (ANOVA *p*-value = 0.092).

Consistent with the trend toward increased NAD(P)H lifetime in 4T07 and 4T1, the Lac/Pyr ratio correlated strongly with increasing NAD(P)H lifetime (R = 0.86, *p* = 0.003) across all measurements (N = 9), suggesting that a large fraction of the enzyme-bound fraction measured by FLIM was associated with aerobic glycolysis (Table 2). The Lac/Pyr ratio also trended with increasing redox ratio (R = 0.63, *p* = 0.071), suggesting a more reduced chemical environment and a shift toward glycolysis in cell lines with a higher Lac/Pyr ratio.

## 4. Discussion

Few studies have combined multiscale modalities to investigate cancer cell metabolism. The current work balances a translational approach that leverages advances in hyperpolarization to directly probe into pyruvate substrate metabolism in concert with FLIM measures in the same cell culture under the same environmental conditions. The complementary information of the multi-modal bioreactor revealed differences in pyruvate utilization and FLIM patterns unique to the highly metastatic and metastatically dormant cell lines. The Lac/Pyr ratio was elevated in both metastatic cell lines (4T1 and 4T07), but less so in the metastatic-dormant cell line (4T07), relative to the non-malignant cell line (67NR). A different pattern was observed for the redox ratio, which was instead significantly higher in the metastatically dormant 4T07 cell line compared to 67NR and qualitatively higher than the 4T1 cell line, a pattern that was largely driven by the FAD intensity, suggesting substantial oxidative metabolism in both 4T07 and 4T1 metastatic cell lines compared to the 67NR cell line. Another study of the same cell lines found that the redox ratio was higher in the 4T1 cell line and comparable to that of the 67NR cell line at normoxia, with a dramatic increase in the redox ratio observed under acute hypoxic conditions, especially in 4T1 and 4T07 compared to the 67NR cell line [25]. Our findings, obtained here under normoxia, showed that 4T07 had a qualitatively higher redox ratio than 4T1 with a significantly higher FAD mean intensity, and that there was a much lower redox ratio in 67NR compared to both 4T1 and 4T07. In our study, the redox ratio, mean and NAD(P)H lifetime tended to be elevated in both metastatic cell lines, suggesting an overall higher fraction of enzyme-bound NAD(P)H. Also in our work, the complementary findings from HP-MRS of elevated Lac/Pyr in the highly metastatic (4T1) cell line in concert with lower FAD intensity suggest greater reliance of the highly metastatic 4T1 cell line on aerobic glycolysis as compared with the metastatic-stable 4T07 cell line, which exhibits an overall elevated metabolism (both glycolytic and oxidative) compared to the non-metastatic cell line (67NR).

For ex vivo or in vitro studies, FLIM is a powerful method with less methodological complexity and greater accessibility than HP-MRS. The complexity of HP-MRS studies is largely due to the need to adapt the bioreactor system to the MRI environment along with the use of the advanced polarizer technology needed to prepare the pyruvic acid solution at 1.4K and then rapidly bring it to 37 degrees prior to injection. This requires complex equipment and carefully timed experimental procedures that are not required for FLIM. However, the HP-MRS method can be used for in vivo patient studies and is being investigated and optimized by several research groups as both a diagnostic and therapy monitoring tool. The application of FLIM for clinical studies remains challenging, but the technique can potentially be performed using cells derived from patient biopsy tissues for clinical assessment. Given the importance of metabolism in cancer, perhaps an ideal workflow can ultimately incorporate both FLIM for ex vivo imaging and HP-MRS for in vivo imaging in a clinical setting.

Moreover, strong correlations of the Lac/Pyr ratio with NAD(P)H lifetime and redox ratio substantially support the interpretation of the Lac/Pyr ratio as indicative of increased aerobic glycolysis. Increased NAD(P)H lifetime is consistent with increased LDH enzyme activity, and increased redox ratio is consistent with increased cellular NADH, both expected with a shift toward aerobic glycolysis. 

The results presented here show preliminary data that indicate different metabolic signatures between non-metastatic (67NR), and metastatic (4T07 and 4T01) breast cancer cell lines. Normal cell metabolism involves oxidative phosphorylation. When oxygen is limited, as during exercise, cells switch to glycolysis and convert most of the glucose to lactate. Cancer cells tend to switch to glycolysis even when oxygen is present (Warburg effect). The switch to glycolysis is an evolved trait that enables tumors to maintain high proliferation despite resistance from their environment and under transient hypoxia during metastasis. It is possible that highly metastatic cells switch to aerobic glycolysis because they require more rapid ATP production and can achieve this through a greater emphasis on glycolytic metabolism. Although glycolysis produces less ATP per mol glucose, it has a higher horsepower (energy produced per volume of enzymes). This upregulation of glycolysis is apparent in the metastatic cell line (4T1) compared to either the non-metastatic (67NR) or dormant (4T07) cell line, indicated by the increased pyruvate to lactate conversion measured by HP-MRS, in concert with lower FAD intensity. 

NAD(P)H and FAD are natural biomarkers that emit endogenous fluorescence and play different roles in the energy metabolism of the cell. NADH is involved in cellular processes such as glycolysis in the cytosol as well as catabolic processes for energy metabolism within the mitochondria. It is interesting that in our study, the FAD intensity was the most significant marker of differences in metastatic potential between the breast cancer cell lines studied. This points to potential differences in mitochondrial metabolism that impact metastatic potential, warranting more careful and direct study. Mitochondria produce reactive oxygen species (ROS) as byproducts of oxidative phosphorylation that play a role in cancer cell signaling, survival, and metastasis. A prior study of the 4T07 and 4T1 cell lines, also under normoxic conditions, showed a non-significant trend toward increased ROS in 4T07 vs. 4T1 [34]. Moreover, rapid cancer cell proliferation can impact mitochondrial fusion, fission, and mutations in mitochondrial DNA (mtDNA), all of which are associated with increased metastatic potential [35,36]. Given the likely involvement of differences in mitochondrial metabolism leading to ROS and associated signaling during rapid proliferation, redox-related signaling pathways between mitochondria and the cell could be targeted for therapy (e.g., reactive cysteine groups on proteins [37]).

There are several important limitations to this work. The sample size is somewhat small, partly due to the throughput in running complex repeated studies with HP-MRS under the controlled cell culture conditions required. One advantage of the FLIM technique is that the workflow and throughput are generally less complex and therefore faster than for HP-MRS. The HP-MRS results can therefore inform us on which FLIM measures might be of greatest relevance to glycolytic vs. oxidative phosphorylation, e.g., FAD and NADH lifetime in our study, to improve sample size and the interpretation of FLIM measurements. Future studies will investigate the HP-MRS results with increased sample size.

Also, our experiment design lacked a negative control group (e.g., normal breast epithelial cells) that could be used to more completely investigate differences in metabolism across the spectrum of normal to malignancy. A negative control in the current work was excluded by design given the translational focus on dormancy vs. metastasis. However, future work should consider this more complete approach. Similarly, this work was conducted under normoxic conditions, with a 5% CO_2_ gas mix. It is likely that different levels of hypoxia will elicit changes in metabolism that are important in the tumor setting. The balance between glycolytic and oxidative phosphorylation is likely to be a dynamic process that is sensitive to oxygen availability, which may explain the discordance between redox ratio results for 4T1 and 67NR cell lines in our study compared to the hypoxic conditions studied in Ref. [25]. Nonetheless, the bioreactor design is fully capable of providing different partial pressure of oxygen (pO2) conditions through the media flow by bubbling different gas mixes prior to circulating through the chamber containing the collagen gel cell culture and should be an area of future work. A fiber optic system for monitoring pO2 (PreSens, Regensburg, Germany) is also built into the design. Future studies will investigate metabolism under different pO2 conditions.

Finally, MCT1 expression may differ by cell line and was not investigated in this work. It is likely that the 4T1 cell line has greater MCT1 expression that may partly drive the greater pyruvate uptake [12]. Therefore, the observed differences may be related to MCT1 expression differences across cell lines and not to metastatic potential via upregulation of glycolysis. Nonetheless, the observed shift to aerobic glycolysis is very likely a combination of pyruvate uptake and greater LDH enzyme activity (and thus greater NAD(P)H utilization) because of the strong correlation between the Lac/Pyr ratio and NAD(P)H lifetime. This finding supports increased enzyme utilization through the greater bound fraction of NADH. Future studies should routinely measure MCT1 expression in cell lines to improve understanding of the balance between enzyme activity and cellular uptake.

## 5. Conclusions

Our results suggest promising differences in metabolism in murine breast cancer cells with differing metastatic potential. The complementary use of HP-MRS and FLIM shows the promise of multi-modal and multiscale imaging for investigating cancer cell metabolism under controlled environmental conditions.

## Figures and Tables

**Table 1:** Summary results of HP-MRS and FLIM comparison.

			NADH		FAD	
Cell Line	Lac/Pyr Ratio	Redox Ratio	Intensity	Lifetime (Tau Mean, ps)	Intensity	Lifetime (Tau Mean, ps)
4T1	0.00293	0.299	613.58	757.8	250.56	751.8
4T1	0.00331	0.343	347.40	810.1	196.88	835.2
4T1	0.00662	0.340	416.14	844.2	238.73	975.8
Mean (SD)	0.00429 (0.0020)	0.327 (0.02)	459.04 (138.2)	804.0 (43.5)	228.7 (28.2)	854.3 (113.2)
4T07	0.00333	0.424	432.05	1000.3	324.82	964.1
4T07	0.0011	0.344	520.70	671.9	288.71	910.8
4T07	0.00096	0.384	438.34	774.9	295.24	971.7
Mean (SD)	0.00180 (0.0013)	0.384 (0.04)	463.70 (49.5)	815.7 (168.0)	302.9 (19.2)	948.9 (33.2)
67NR	0	0.273	464.36	749.8	134.70	813.2
67NR	0	0.263	414.45	339.5	160.55	396.9
67NR	0	0.203	448.20	420.1	113.11	935.1
Mean (SD)	0 (0)	0.246 (0.04)	442.33 (25.5)	503.1 (217.4)	136.1 (23.8)	715.1 (282.2)

**Table 2:** Spearman correlation values for HP-MRS and fluorescence microscopy measures (* indicates significant association; ^+^ indicates a trend).

Comparison HP-MRS Lac/Pyr Ratio vs.	Spearman Correlation Coefficient	*p*-Value
Redox Ratio	0.63	0.071 ^+^
NADH Intensity	−0.25	0.51
NADH Lifetime	0.86	0.0026 *
FAD Intensity	0.59	0.092
FAD Lifetime	0.49	0.18

## Data Availability

The raw data supporting the conclusions of this article will be made available by the authors on request.
